# SSR-Based Analysis of Genetic Diversity and Structure of Sweet Cherry (*Prunus avium* L.) from 19 Countries in Europe

**DOI:** 10.3390/plants10101983

**Published:** 2021-09-23

**Authors:** Teresa Barreneche, María Cárcamo de la Concepción, Marine Blouin-Delmas, Matthew Ordidge, Hilde Nybom, Gunars Lacis, Daina Feldmane, Jiri Sedlak, Mekjell Meland, Hedi Kaldmäe, Kersti Kahu, Zsuzsanna Békefi, Sanda Stanivuković, Gordana Đurić, Monika Höfer, Martin Galik, Elisabeth Schüller, Andreas Spornberger, Sorina Sirbu, Pavlina Drogoudi, Ana Cristina Agulheiro-Santos, Ossama Kodad, Aleš Vokurka, Marc Lateur, Felicidad Fernández Fernández, Daniela Giovannini, José Quero-García

**Affiliations:** 1 INRAE, University of Bordeaux, UMR BFP, 33882 Villenave d’Ornon, France; teresa.barreneche@inrae.fr; 2 Faculty of Engineering and Science, Natural Resources Institute, University of Greenwich, Chatham, Kent ME4 4TB, UK; Maria.CarcamoDeLaConcepcion@greenwich.ac.uk; 3 INRAE, Unité Expérimentale Arboricole, Domaine de la Tour de Rance, 47320 Bourran, France; marine.delmas@inrae.fr; 4 Department of Crop Science, School of Agriculture, Policy and Development, University of Reading, Reading RG6 6EU, UK; m.ordidge@reading.ac.uk; 5 Balsgård-Department of Plant Breeding, Swedish University of Agricultural Sciences, Fjälkestadsvägen 459, 29194 Kristianstad, Sweden; Hilde.Nybom@slu.se; 6 Institute of Horticulture, Graudu 1, LV-3701 Dobele, Latvia; gunars.lacis@llu.lv (G.L.); daina.feldmane@llu.lv (D.F.); 7 Research and Breeding Institute of Pomology Holovousy Ltd., Holovousy 129, 508 01 Hořice, Czech Republic; Jiri.SEDLAK@vsuo.cz; 8 NIBIO Ullensvang, The Norwegian Institute of Bioeconomy Research, Ullensvangvegen 1005, N-5781 Lofthus, Norway; mekjell.meland@nibio.no; 9 Polli Horticultural Research Centre, Institute of Agricultural and Environmental Sciences, Uus 2, 69108 Polli, Estonia; hedi.kaldmaee@emu.ee (H.K.); kersti.kahu@emu.ee (K.K.); 10 National Agricultural Research and Innovation Centre Gödöllő, H-1223 Budapest, Hungary; bekefi.zsuzsanna72@gmail.com; 11 Institute for Genetic Resources, University of Banja Luka, Bulevar vojvode Petra Bojovica 1A, 78000 Banja Luka, Bosnia and Herzegovina; sanda.stanivukovic@igr.unibl.org (S.S.); gordana.djuric@agro.unibl.org (G.Đ.); 12 Federal Research Centre for Cultivated Plants, Institute for Breeding Research on Fruit Crops, Julius Kühn Institute, Pillnitzer Platz 3a, 01326 Dresden, Germany; monika.hoefer@julius-kuehn.de; 13 NPPC, Výskumný ústav Rastlinnej Výroby–VÚRV, Research Institute of Plant Production–RIPP, Bratislavská 122, 921 68 Piešťany, Slovakia; martin.galik@nppc.sk; 14 Division of Viticulture and Pomology, University of Natural Resources and Life Sciences, Vienna Gregor-Mendel-Strasse 33, 1180 Vienna, Austria; elisabeth.schueller@gmail.com (E.S.); andreas.spornberger@boku.ac.at (A.S.); 15 Research Station for Fruit Growing, 175 Voinesti, RO707305 Iasi, Romania; office@pomicolaiasi.ro; 16 Hellenic Agricultural Organization ‘DEMETER’, Department of Deciduous Fruit Trees, Institute of Plant Breeding and Genetic Resources, 38 RR Station, 59200 Naoussa, Greece; drogoudi@otenet.gr; 17 Mediterranean Institute for Agriculture, Environment and Development & Departamento de Fitotecnia, Escola de Ciências e Tecnologia, Universidade de Évora, Pólo da Mitra, Ap. 94, 7006-554 Évora, Portugal; acsantos@uevora.pt; 18 Département Arboriculture Arboriculture Fruitière Viticulture Ecole Nationale d’Agriculture de Meknès, B.P. S/40, Meknès 50000, Morocco; osama.kodad@yahoo.es; 19 Department for Plant Breeding, Genetics and Biometrics, Faculty of Agriculture, University of Zagreb, Svetošimunska 25, HR-10000 Zagreb, Croatia; avokurka@agr.hr; 20 CRA-W, Centre Wallon de Recherches Agronomiques, Plant Breeding & Biodiversity, Bâtiment Emile Marchal, Rue de Liroux, 4-5030 Gembloux, Belgium; m.lateur@cra.wallonie.be; 21 Genetics, Genomics and Breeding Department, NIAB EMR, New Road, East Malling, Kent ME19 6BJ, UK; Felicidad.Fernandez@emr.ac.uk; 22 CREA-Research Centre for Olive, Fruit and Citrus Crops, via la Canapona 1 bis, 47121 Forlì, Italy; daniela.giovannini@crea.gov.it

**Keywords:** *Prunus avium*, SSR, genetic diversity, population structure, genetic resources, breeding

## Abstract

Sweet cherry (*Prunus avium* L.) is a temperate fruit species whose production might be highly impacted by climate change in the near future. Diversity of plant material could be an option to mitigate these climate risks by enabling producers to have new cultivars well adapted to new environmental conditions. In this study, subsets of sweet cherry collections of 19 European countries were genotyped using 14 SSR. The objectives of this study were (i) to assess genetic diversity parameters, (ii) to estimate the levels of population structure, and (iii) to identify germplasm redundancies. A total of 314 accessions, including landraces, early selections, and modern cultivars, were monitored, and 220 unique SSR genotypes were identified. All 14 loci were confirmed to be polymorphic, and a total of 137 alleles were detected with a mean of 9.8 alleles per locus. The average number of alleles (N = 9.8), PIC value (0.658), observed heterozygosity (H_o_ = 0.71), and expected heterozygosity (H_e_ = 0.70) were higher in this study compared to values reported so far. Four ancestral populations were detected using STRUCTURE software and confirmed by Principal Coordinate Analysis (PCoA), and two of them (K1 and K4) could be attributed to the geographical origin of the accessions. A N-J tree grouped the 220 sweet cherry accessions within three main clusters and six subgroups. Accessions belonging to the four STRUCTURE populations roughly clustered together. Clustering confirmed known genealogical data for several accessions. The large genetic diversity of the collection was demonstrated, in particular within the landrace pool, justifying the efforts made over decades for their conservation. New sources of diversity will allow producers to face challenges, such as climate change and the need to develop more sustainable production systems.

## 1. Introduction

Agriculture will face crucial challenges in the near future, including the need for substantially increased food production in order to satisfy 8 billion people. The impact of climate change is already causing a reduction in agricultural production at a global scale while food demand increases steadily. Experts from the Intergovernmental Panel on Climate Change predict that this situation will worsen in the coming years [1]. Diversity in plant material may help to alleviate climate risks by enabling farmers to adapt crops and ensure food production. During recent decades, significant efforts have been devoted to the collection and conservation of worldwide plant genetic resources [2]. The European Cooperative Program for Plant Genetic Resources Networks (ECPGR, https://www.ecpgr.cgiar.org/ accessed on 9 September 2021), established in1980, is funded by European countries with the precise scope of ensuring long-term conservation and facilitating the utilization of Plant Genetic Resources for Food and Agriculture (PGRFA) in Europe. The ECPGR network is organized in thematic and crop Working Groups, including the Prunus WG (https://www.ecpgr.cgiar.org/working-groups/prunus accessed on 9 September 2021). From 2003, the European Search Catalogue for Plant Genetic Resources (EURISCO, https://www.ecpgr.cgiar.org/resources/germplasm-databases/eurisco-catalogue/ accessed on 9 September 2021), coordinated and funded by ECPGR, provides information at the accession level of PGRs conserved ex situ and in situ [3]. In addition, in order to provide access to characterization and evaluation data on accessions of the *Prunus* collections, in 2012, the *Prunus* WG developed the European *Prunus* DataBase (EPDB, http://www.bordeaux.inra.fr/euprunusdb accessed on 9 September 2021). More recently, in the frame of the EU.Cherry ECPGR project (https://www.ecpgr.cgiar.org/working-groups/prunus/eucherry accessed on 9 September 2021), a collaborative action focused on sweet cherry, the WG has embarked on updating, documenting, and communicating the patrimonial richness of cultivated *Prunus avium* L. in Europe, aiming to connect information of the EURISCO and EPDB databases.

Sweet cherry is a major fruit species in Europe, and it is an early summer crop, very much appreciated by consumers for its organoleptic qualities (color, firmness, and taste). The beneficial health effects have attracted attention as sweet cherry is rich in polyphenols [4,5] in addition to the presumed nutraceutical value [6]. Moreover, in many European countries, where fruit production takes place mainly on small farms, sweet cherry represents the first significant source of income after the winter. Cultivation of sweet cherry has long been thought to have initiated in Greece [7], from where it later spread to the whole continent. Over the centuries, a multitude of cherry landraces has emerged in many European regions. Landraces have invariably been selected for suitability to the pedoclimatic environment as well as the food habits and customs of local users. However, the selection of modern cultivars at the end of the 19th and the beginning of the 20th centuries initiated the decline of cherry landraces all over Europe. More recently, this trend has continued due to the promotion of more intensive monocropping practices and the focus on a few high-performing and economically profitable modern cultivars. As a result, entire European traditional cherry cultivation regions and their associated landraces have disappeared, often replaced by a few sweet cherry new varieties or, even worse, by crops more economically profitable. For instance, in the ‘Entre deux Mers’ region, in the Southwest of France, the cherry trees of local cultivars were removed to favor the extension of vineyards [8]. Political developments have also affected cherry cultivation; for instance, the change in regime in Romania at the end of the 1980s provoked drastic changes in terms of land ownership and resulted in the abandonment of large areas of agricultural land (Budan, pers. comm.). The wars in the former Yugoslavia in the 1990s similarly reduced the land area devoted to traditional cherry production, such as the famous ‘Maraska’ sour cherry in Croatia (Vokurka, pers. comm.). An economic model based on the utilization of local cultivars has been developed in some regions with varying levels of success, such as the ‘Valle del Jerte’ region in Southwest Spain (https://cerezadeljerte.org/en/the-picota-of-jerte accessed on 9 September 2021), and the ‘Itxassou’ village in Southwest France (http://www.cerise-itxassou.com/ accessed on 9 September 2021). European public institutes and non-governmental organizations have also been working for decades to collect and preserve ex situ cherry landraces and modern cultivars (see EURISCO, EPDB and [9]).

Currently, molecular markers (RAPD, AFLP, ISSR, SSR, and SNP) are being used to analyze the genetic diversity of European landraces and identify putative synonyms in a whole range of fruit tree species and other vegetatively propagated crops [10]. Studies on sweet cherry genetic diversity involving landraces of different European countries, such as Austria [11], Bosnia Herzegovina and Croatia [12], Czech Republic [13], France [14,15], Germany [16], Greece [17], Italy [18,19,20,21], and Lithuania [22], have been published in the last decade. However, most of these studies have focused on a limited number of landraces from each country or even from a region within a country. Exploration of the large, sweet cherry diversity in Europe could help to tackle the crucial challenges currently facing European producers, such as climate change and the invasion of new pathogens. Therefore, partners of the EU.Cherry project and of the COST cherry network (https://www.bordeaux.inra.fr/cherry/ accessed on 9 September 2021) recently joined forces to analyze a large set of European sweet cherry landraces together with some popular old and modern cultivars. One of the long-term aims of this initiative is to define a first European *core-collection*, highly representative of sweet cherry diversity still available in Europe. Here we investigated accessions from 19 countries using 18 microsatellite loci, with the following objectives: (i) assess genetic diversity; (ii) analyze patterns of population structure; (iii) and identify germplasm redundancies.

## 2. Results

### 2.1. Genotype Redundancy in the European Sweet Cherry Collection

**Figure 1:**
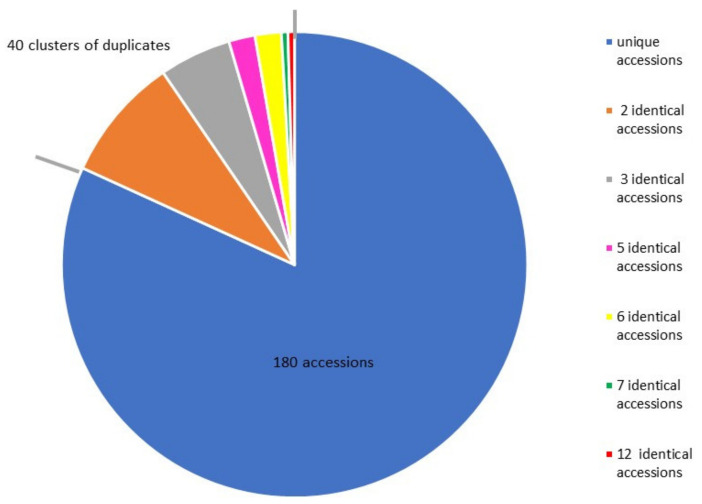
Distribution of SSR genotypes in the whole collection (314 accessions).

Of the 18 SSR loci scored, four (EMPa015, EMPaS01, EMPaS10, and UDP96-005) showed inconsistent allele patterns, and so a subset of 14 loci was used for further analysis. Three hundred and fourteen accessions presented allelic patterns corresponding to diploid genotypes, whereas 10 accessions presented additional alleles in various loci suggesting that they were tetraploid and most likely belonging to sour cherry (*P. cerasus*) instead of sweet cherry. These were discarded from further analysis. Among the 314 diploid accessions, a total of 180 accessions represented genotypes that were only identified once. The remaining 134 accessions could be allocated to 40 groups based on having identical genotypes. These groups varied in size from groups with only two accessions each (19 groups) to a group containing 12 accessions. Some redundancies were found within samples from the same national collection, and some were found between collections (Figure 1 and Table S1).

The proportion of unique relative to duplicated genotypes within samples from differing national collections varied substantially, ranging from 94% duplicated genotypes in the samples from the Moroccan collection to 9% duplicated genotypes in the samples from the Latvian collection (Figure S1). The largest numbers of unique genotypes were obtained from France and Latvia (amounting to 9.6% and 9.2% of the whole collection, respectively), whereas the samples from Morocco contributed only one unique genotype. One accession, generally held under the most well-known cultivar name (assuming trueness to type), was chosen in each group to represent the group genotype in the data set (e.g., ‘Bigarreau Hâtif Burlat’ for group 36, ‘Hedelfinger’ for group 39, and ‘Ferrovia’ for group 40). A total of 220 accessions with unique SSR genotypes, therefore, remained for further studies (Table S2).

### 2.2. Genetic Diversity Estimation by 14 SSR Loci

All 14 loci were confirmed to be polymorphic, and a total of 137 alleles were detected with a mean of 9.8 alleles per locus, ranging from 5 alleles for EMPa002 to 15 alleles for BPPCT034. PIC values ranged from 0.358 (EMPa017) to 0.838 (EMPaS06) with an average of 0.658. Observed heterozygosity (Ho) varied from 0.350 (EMPa017) to 0.950 (BPPCT034) with a mean value of 0.709. Expected heterozygosity (He) varied from 0.380 (EMPa017) to 0.850 (EMPaS06) with a mean value of 0.699. The inbreeding coefficient Fis ranged from −0.17 (BPPCT034) to 0.08 (EMPa017), six loci showed a slight excess of heterozygotes, but only BPPCT034 showed a statistically significant deviation from the Hardy–Weinberg equilibrium. Thirty-nine percent of the alleles occurred in less than 1% of the accessions (and were classified as rare alleles); from 8 alleles in EMPa018 to only 1 in CPSCT038 and EMPaS02 (Table 1).

Most of the rare alleles were found in accessions classified as landraces (69%), while early selections (11%) and modern cultivars (20%) reported fewer (Table S3). The Slovakian accessions had the highest relative number of rare alleles. Private alleles were found in all loci except EMPaS02. The Italian landrace ‘Malizia’ contained the highest number of private alleles (6 out of 24 alleles in total), whereas two private alleles were found in ‘Žalanka’, ‘Saint Georges’, and ‘Galata’ from the Czech Republic, France, and Romania, respectively. Another 12 accessions had only one private allele (Figure S2). The population genetics analyses were repeated on two separate sets of accessions: landraces (L), and early selections and modern cultivars (ES-MB) (Table S4). The mean number of alleles per locus was higher in L (9.1) compared to ES-MB (7.3). Similarly, the number of rare alleles, number of private alleles, and H_e_ were slightly higher in L, whereas H_o_ was slightly lower. (Table 2). In ES-MB, more loci showed an excess of heterozygotes than in L, with only two (BPPCT034 and EMPa002) presenting a statistical deviation from the Hardy–Weinberg equilibrium (Table S4).

### 2.3. Population Structure

**Figure 2:**
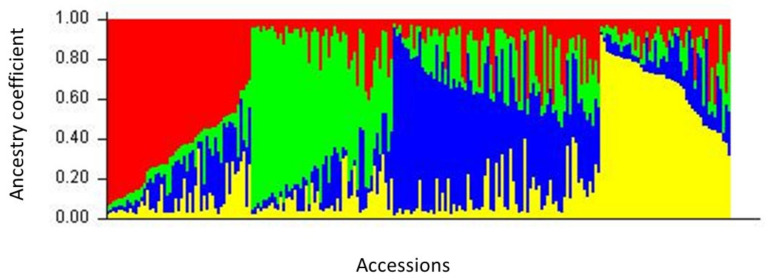
Inferred population structure of the collection using STRUCTURE software. Bar plot of individual ancestry proportions for the genetic clusters inferred using STRUCTURE (K = 4) and the 14 SSR data. Individual ancestry proportions (q values) are sorted within each cluster. The admixture model, independent frequencies, 100,000 burn-in iterations, 100,000 Markov Chain Monte Carlo iterations were used for this analysis. The different clusters are shown in red (cluster 1), in green (cluster 2), in blue (cluster 3), and in yellow (cluster 4).

The most likely number of populations determined by Bayesian analysis was K = 4 (Figure S3a,b). Using a threshold of membership >80%, only 46 out of 220 genotypes could be assigned to a population at K = 4 (Figure 2, Table S5).

Clusters 1, 2, and 4 contained 14, 14, and 12 accessions, respectively, while cluster 3 was smaller (containing 6 accessions). The number of admixed genotypes was 174 (89% of the total). However, by lowering the membership threshold to 55%, 13% of the previously admixed accessions could be assigned to cluster 1, 13 % to cluster 2, 21% to cluster 3, and 12% to cluster 4. More than a third, i.e., 78 accessions, still remained as admixed since they had a membership coefficient below 0.55 (Table S6). When using the 80% membership threshold, cluster 1 contained most of the Italian accessions, cluster 4 contained accessions maintained in the Baltic countries (73% from Estonia and 26% from Latvia), whereas cluster 2 included well-known landraces from different countries. When considering the 55% membership threshold, 78% of the accessions from the Italian collection clustered in K1, and the proportion of accessions maintained in Estonian and Latvian collections reached respectively 100% and 62% in K4. The distribution within the four STRUCTURE-defined populations of the accessions, according to their country of origin, suggested a gradient from Northeast to Southwest of Europe (Figure S4). Allele frequency divergence between the 4 clusters calculated with STRUCTURE (Table 3) showed a high proximity of clusters K2 and K4 (0.0996) and clusters K1 and K2 (0.1004). Cluster K3 diverged considerably when compared to cluster K4 (0.1920) and cluster K1 (0.1835), although less from cluster K2 (0.1178) (Table 3). In terms of allelic diversity variation, cluster K2 appeared as the largest with a mean distance between individuals of 0.75, while clusters 1, 3, and 4 were roughly similar, and the mean distance between accessions in each cluster was respectively 0.53, 0.55, and 0.58 (Table S7).

**Figure 3:**
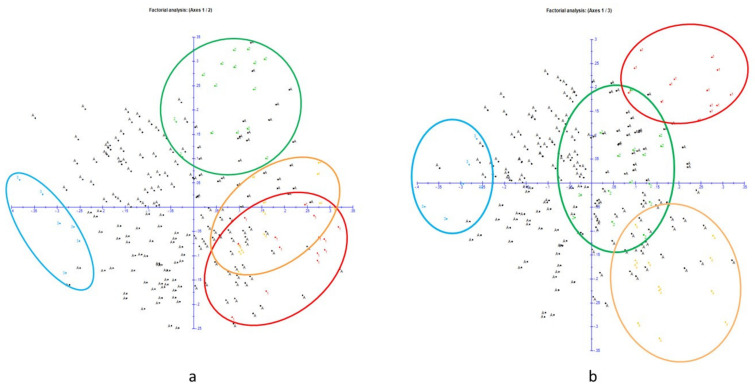
Principal Coordinates Analysis (PCoA) of the 14 SSR across the 220 sweet cherry accessions. The STRUCTURE groups K1 (red), K2 (green), K3 (blue), K4 (yellow), and A (admixed in black) are shown. First and second components (**a**) and first and third components (**b**) of the PCoA analyses are shown. Axes 1, 2, and 3 explained respectively 7.64%, 5.83%, and 4.71% of the variation.

The results obtained with STRUCTURE were confirmed by the representation of accessions in PCoA analysis. Accessions could be divided into four groups corresponding to the populations previously identified, with the admixed accessions dispersed among the rest (Figure 3a,b).

In both PCoA plots, cluster K3 separated clearly from the three others. In terms of allelic diversity variation, cluster K2 appeared as the largest.

**Figure 4:**
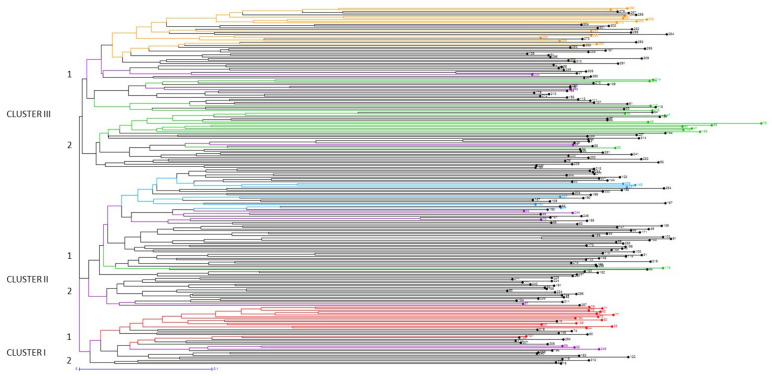
Neighbor-joining tree of the 220 sweet cherry accessions. Accessions were colored according to their membership to STRUCTURE populations (K1 = red, K2 = green, K3 = Blue, K4 = yellow, admixed = black). Some reference early selection and modern breeding cultivars were colored in purple. Accession number is indicated in the figure; for correspondence with accession name, see Table S8.

A Neighbor-joining (N-J) tree based on a simple matching dissimilarity matrix between the 220 accessions of the European collection separated the sweet cherry accessions into three main clusters and six subclusters (Figure 4, Table S8). Nevertheless, since the distances separating these clusters were very short, the stability of this structure has to be considered cautiously. However, accessions were grouped in Table S8 in different subclusters and subgroups for ease of reading. Accessions from the four identified STRUCTURE populations clustered roughly together within the N-J tree.

Cluster I consisted of a group of 36 accessions, which were mainly early selections and modern breeding cultivars (63% of total cluster I accessions). All the accessions forming the STRUCTURE K1 population were present in this cluster, including most of the Italian accessions, which were mainly landraces. Among the latter, ‘Duroncino di Cesena’ showed the greatest diversity. ‘Mora di Cazzano’, another Italian landrace maintained in the French collection, appeared related to ‘Adriana’, which is one of its offspring. Associated with them was ‘Bigarreau Camus de Venasque’, which is a French landrace from Southwest France near the Italian border. The rest of the cluster consisted mainly of modern cultivars, as well a popular old cultivars, the early selection ‘Emperor Francis’. Four Romanian cultivars (‘Andrei’, ‘Ludovic’, ‘Daria’, and ‘Bucium’) clustered relatively close to each other; indeed, they all derive from crosses with ‘Boambe de Cotnari’ as parent. ‘Ludovic’ and ‘Bucium’ are offspring of the well-known Canadian cultivar ‘Van’, which also clustered in the same region of the dendrogram. The remaining cultivars were modern ones from different Northeast European countries, most of them having at least a German accession as parent.

Cluster II grouped 85 accessions; among them, 62% were landraces and 38% early selection and modern breeding cultivars. Accessions in cluster II were mainly from North, Northwest, and Central Europe. Cluster II comprised most of the admixed accessions identified by Bayesian analysis, as well as all the accessions found in the K3 population. In the upper part of this cluster, a group of ‘guignes’, including ‘Ham Green Black’, ‘Guigne du Champ de l’air’, ‘Karting Fran Djupekas’, ‘Goodnestone Black’, and ‘Guigne Presgaux 2’ were closely related. All the Belgian landraces in this study except ‘Bigarreau Ghijssens’ and ‘Rouge dorée’ were found in this cluster, more or less close to ‘Cerise Proces’. All the accessions from the K3 STRUCTURE population were grouped in this part of the dendrogram, and they were in majority landraces from distant countries, such as Belgium (‘Cerise Proces’), Hungary (‘Torbágyi Késői’ and ‘Solymári Gömbölyű’), and Norway (‘Osabaer’). The French early selection (‘Bigarreau Marmotte’) was grouped close to several well-known early selections and modern cultivars, such as ‘Napoleon’ (the accession maintained in the French collection), ‘Hedelfinger’, ‘Bing’, its offspring ‘Sue’, and the Japanese cultivar ‘Nanyo’, which results from ‘Napoleon’ open pollination. Other well-known European modern breeding cultivars, such as the Italian ‘Ferrovia’, the Romanian ‘Uriase de Bistrita’, and the Hungarian ‘Kavics’ clustered closely, and the two latter having ‘Gemersdorfer’ as parent. The lower part of cluster II included numerous landraces from central European countries, such as one landrace from Switzerland maintained in the French collection, ‘Hâtive de Bâle’, four landraces from Austria, all Bosnian landraces and the only studied Croatian landrace, ‘Okićka’, but also landraces from North and Northwest Europe (Norway and Great Britain). Several early selections and modern cultivars could, though, be found in these subclusters. For instance, German accessions ‘Knauffs Schwarze’, ‘Kassins Frühe’, ‘Nafrina’, and ‘Werder’ were clearly grouped, the last one belonging to the Norwegian collection and supposed to be one of the parents of ‘Nafrina’. The other early selections or modern cultivars were mostly originated from Germany, Romania, or Sweden. Among them, we can cite another accession named ‘Napoleon’, maintained in the Norwegian collection, and the well-known German cultivars ‘Schmidt’ and ‘Regina’.

Cluster III was the largest one with 99 accessions. It included all the accessions of the K4 and K2 populations identified by Bayesian analysis. The upper part was constituted mainly by early selection and modern breeding cultivars. It comprised all accessions of the K4 STRUCTURE population, which are mainly maintained in the Estonian and Latvian collections and originated from or were released in Northeastern Europe (Estonia, Lithuania, Latvia, and Russia). Many of these cultivars share the genitor ‘Leningradskaya Chornaya’ in their pedigree. All the Ukrainian modern bred cultivars were also found in this cluster which has the German early selection cultivar ‘Drogans Gelbe Knorpelkirsche’ as common parent. In the lower part of cluster III all accessions of the K2 STRUCTURE population were grouped, with the exception of the Romanian cultivar ‘Galata’, present in cluster II. Among these, the early selection cultivars ‘Early Birchenhayes’ and ‘Burcombe’ from Great Britain, the Greek landrace ‘Tragana Edessis’, and the Romanian modern cultivar ‘Maxut’ were clearly separated from a large group of accessions which were all landraces. There were several groupings of accessions from the same geographical regions, e.g., early selections from Great Britain, Greek landraces along with the modern cultivar ‘O.T.E.A.’, a large group of French landraces integrating the K2 STRUCTURE population and three cultivars from Latvia and Lithuania. The last accession from STRUCTURE population K2, ‘Lisboeta’, was grouped in the dendrogram along with landraces from Bosnia and Herzegovina and France, as well as with the well-known French early selections ‘Bigarreau Hâtif Burlat’ and ‘Bigarreau Moreau’. The American modern breeding cultivar ‘Glacier’, resulting from the hybridization of cultivars ‘Stella’ and ‘Bigarreau Hâtif Burlat’, was closely clustered. Finally, the accessions grouped within the lower part of cluster III presented a high geographical diversity (9 countries involved) and a predominance of landraces.

Overall, considering the different clusters, in terms of genetic diversity, the most original accessions were ‘Duroncino di Cesena’, ‘Bigarreau Camus de Venasque’, ‘Hedemora’, ‘Techlovicka’, ‘Alica’, ‘Galata’, ‘Draganele de Pitesti’, ‘Malizia’, ‘Durette’, ‘Dvijla Cerna’, ‘Maardu Maguskirss’, and ‘Radica’. The most distant accession of the whole study was the Italian landrace ‘Malizia’ within Cluster III.

## 3. Discussion

### 3.1. Genotype Redundancy in the Sweet Cherry Collection

One of the goals of this study was to contribute to the development of a joint European germplasm collection, which is easy to use by sweet cherry breeders. Among the 314 accessions initially collected, 40 groups with shared genotypes were found. Redundancies occurred both within and between samples of accessions from the national collections. A range of errors was indicated, including incorrect passport data due to spelling mistakes in accession names translation, in particular when transcribing accessions names from the Cyrillic to Latin alphabet: e.g., ‘Iput’ (Estonian collection) and ‘Iputj’ (Latvian collection) or to mislabeling, e.g., ‘Fryksås’ (Swedish collection) which was deemed to match ‘Gårdebo’ and is believed to be a mislabeled accession. This latter finding agrees with the alignment against the Swedish national dataset, which also associated these two last accessions with accessions of the name ‘Wils Frühe’ in both Germany and Great Britain collections [9]. Accessions with the same (or very similar) names sometimes did have the same SSR genotype, e.g., ‘Tavora A’ (aINIAV-101) = ‘Tavora VR’ (INIAV-106) in the Portuguese collection. By contrast, ‘Leningradskaja Chernaja’ from the Estonian collection and ‘Leningradskaja Chornaya’ from the Latvian collection showed different SSR profiles despite the names being supposedly the transcription of the same Cyrillic name. Other cases where the same name was associated with different SSR genotypes, most likely due to mislabeling or homonymy, included ‘Napoleon 1A’ (accession Mor013 in the Moroccan collection) and ‘Napoleon’ (accession FRA057-036 in the French collection). A third accession (505) labeled ‘Napoleon’ in the Norwegian collection showed a unique SSR profile, which could agree with historical records that report two ‘Napoleon’ types being disseminated in Europe in the past, the true-to-type ‘’blush-type’ fruit (FRA057-036) and a cultivar with red fruit. The Moroccan collection included two accessions under the name of the Canadian modern cultivar ‘Van’: Mor016 labeled as ‘Van 1Q’ and Mor017 labeled as ‘Van’. The first had the same SSR profile as true-to type ‘Van’ maintained in the French collection (as well as accessions under the names ‘CA’ and ‘VN’ from Morocco and ‘Maringa’ from Portugal), while the second belonged to the largest group of redundant accessions in our analysis (group 40). Some synonymy represented within this group has already been reported, such as ‘Ferrovia’ = ‘Badacsony’ [16], and the group was also the largest group of matching entries identified in a recent study [10]. Another example consists of ‘Morangal’ = ‘Grosse Rouge de la Faculté’ = ’Crveni Hrušt’ = ‘Boambe de Cotnari’ = ‘Büttners Rote Knorpelkirsche’ = ‘Emperor Francis’, which differ in the country of origin, status, and period of release but still were surprisingly similar, as previously reported for part of them [15]. Despite being sampled in different national collections, accessions with the same name showed identical SSR profiles in several cases, e.g., ‘Früheste der Mark’ (from German and Norwegian collections), ‘Burlat’ and ‘Bigarreau Hâtif Burlat’ (from Moroccan and French collections), and ‘Early Rivers’ (from British and Norwegian collections). For example, twenty groups of potential synonyms were unique to our study, while the grouping of an accession under the name ‘Bigarreau Hâtif Burlat’ from France with one under the name ‘Chalkidos’ in Greece (and accessions under the names ‘Bah’, ‘BGh’, ‘BN’, and ‘Burlat 1R’ from Morocco) in our group 36 agrees with the aligned data in a recent study [9]. Some accessions with major traits (pomological or phenological) reflected in their names were apparently synonymous, such as the early-ripening ‘Aprilska’ and ‘Früheste der Mark’, the black-fruited ‘Abbesse de Mouland’, ‘Bigarreau Noir’, and ‘Bigarreau Noir Hâtif’, and the yellow-fruited ‘Branca’, ‘Grosse Blanche de Verchocq’, ‘Kirsche Kierling’, ‘Drogans Gelbe Knorpelkirsche’, ‘Bigarreau Jaune Tardif’, and ‘Dzintars’ which appeared also grouped with a French accession under the name ‘Guigne Jaune Donissen’ in a recent study [9].

### 3.2. Genetic Diversity Estimation

Average number of alleles (N = 9.8), PIC value (0.658), observed heterozygosity (H_o_ = 0.71), and expected heterozygosity (H_e_ = 0.70) were higher in this study compared to values reported in most other sweet cherry studies [11,13,18]. Exploration of two germplasm collections from Southern and Central Italy revealed a mean expected heterozygosity H_e_ = 0.56 and 6.5 alleles per locus [18]. A survey of Czech cultivars studied with 16 SSR reported a mean expected heterozygosity H_e_ = 0.59 and 4.3 alleles per locus [13]. Assessing the genetic diversity of 20 Lithuanian sweet cherry accessions showed slightly higher values for H_o_ and H_e_, respectively (0.68 and 0.66) [22]. A study of Austrian sweet cherry varieties resulted in similarly high values as in the Lithuanian study: H_o_ = 0.66 and H_e_ = 0.64 [11]. All these results agreed with values of heterozygosity reported by other authors for sweet cherry: 0.49 [23], 0.50 [24], 0.66 [25], 0.59 [26], 0.61 [27]. More recently, a study involving a large sample of sweet cherry landraces from the Campanian region in South Italy and some foreign cultivars using 15 SSR still highlighted slightly lower values than those obtained in this work (N = 7.33; PIC = 0.561; H_o_ = 0. 62; H_e_ = 0.63) [21]. Similar values as those of the Italian study were found when evaluating sweet cherry genetic resources (123 accessions of landraces and cultivars) from different counties maintained in Czech Republic (H_o_ = 0.61; H_e_ = 0.65) [28]. Likewise, similar values (H_o_ = 0.63; H_e_ = 0.63) were obtained when characterizing 207 accessions (landraces and modern bred cultivars) from a French germplasm collection [14]. With the exception of the latter two, all these studies concerned either small national scale samples or large sampling of local cultivars. Noting that the samples included in our analysis were relatively small subsets from a range of larger national collections, the higher H_o_ and H_e_ values obtained in this work possibly reflect the large geographical sampling range. In addition, they reflect the high genetic diversity among the European accessions forming the collection, and similar values were found from the analysis of a larger group of accessions aligned across more complete samples from a subset of the national collections [9].

The differences for H_o_, H_e_, % of rare alleles, and inbreeding coefficient (Fis) between landraces and either early selection or modern breeding cultivars agreed with the loss of diversity associated with breeding [14,15,29]. These differences further highlight the potential of landraces as a source of novel genetic diversity in sweet cherry breeding. Comparing our results with similar studies [14], a higher genetic diversity was found, either within the landraces or the selected cultivars subsets, reflecting again the richness of the European collection and its large geographical distribution range.

### 3.3. Genetic Structure among European Accessions

The two different approaches (STRUCTURE and PCoA) used here to analyze the structure of the European sweet cherry accessions provided similar results. This clustering reflected, to some extent, the geographical origin of the accessions. Thus, population K1 contained most of the Italian accessions, and some of these (‘Cornetta’, ‘Duroncino di Cesena’, ‘Durone Nero II’, ‘Fiore’, ‘Morandina’, and ‘Mora di Cazzano’) clustered together also in a previous study [21]. Other accessions, such as ‘Malizia’, ‘Ferrovia’, and ‘Gemella’, were placed in a different group or were admixed, corroborating again results obtained here (‘Malizia’ in K2 population and ‘Gemella’ and ‘Ferrovia’ admixed) [21]. On the opposite, ‘Morena’ was clearly placed within the population K1 in our work (membership coefficient = 0.91) while it appeared admixed in the former study. A chance seedling from the Czech Republic was also found in the K1 population, which stands as a reference for fruit quality, cracking tolerance, and late ripening [30]. The French landraces ‘Bigarreau Coeur de Pigeon tardif’, ‘Blancale Precoce’, ‘Durette’, ‘Saint Georges’, and ‘Xapata’ and the Spanish ‘Cristobalina’ clustered together in the K2 population, in agreement with previous studies mainly focused on INRAE (French National Research Institute for Agriculture Food and Environment) germplasm collections [14,15]. Associated with them were some of the accessions showing the higher number of rare alleles (and in some cases private alleles), such as ‘Brdarka 245’, ‘Maxut’, ‘Galata’, and ‘Malizia’. The latter being the most original in our analysis with seven rare alleles, among them six being private. The K3 population contained only six accessions but appeared as more divergent when compared to the other three clusters. Furthermore, 21% of the admixed accessions of the whole data set showed at least 55% of their genome in K3, suggesting the significance of this population. Finally, the K4 population gathered together the accessions of Northeast Europe and Russia (maintained in Baltic countries Latvia and Estonia). Most of them were modern breeding cultivars released by Estonian and Russian breeding programs and often involving ‘Leningradskaya Chernaya’ as one parent (Feldmane D, pers. comm.). Mainly all the Estonian and the Russian accessions and some Latvian ones (‘Aleksandrs’, ‘Iedzenu Dzeltenais’, and ‘Paula’) had at least more than 55% of their genome in population K4. While the remaining Latvian admixed accessions showed more than 55% of their genome in K2 (‘Talsu 1, ‘Strazdes Agrais’, and ‘Indra’) or broke out in the four populations. Conversely, most of the Ukrainian accessions were admixed and were not related to the K4 population. As reported for apple [31], the genetic composition of accessions from each national collection (including admixed accessions with membership coefficient > 0.55) suggested a potential gradient from the Northeast to Southwest of Europe. However, this trend should be tested using a more exhaustive sweet cherry European sample. In the light of the evolutive history of *Prunus avium* L. in Europe and our findings in relation to the numbers of rare alleles in Greek samples, the genetic resources of some essential regions including Greece, Croatia, Serbia, and Slovakia were underrepresented in our study. Moreover, geographical regions, including Bulgaria and Spain, were completely absent, and it would be valuable to consider these in further analyses.

Understanding the genetic relationships among accessions from different countries’ collections is crucial to define a European core collection. The N-J tree grouped the 220 sweet cherry accessions within three main clusters not necessarily related to the accession geographical origin. However, at least some clusters were associated with accession country of origin, i.e., Italian accessions in cluster I, German early selection cultivars in cluster II, and accessions from Northeast countries (Latvia, Estonia, Lithuania, and Russia) in cluster III. So far, few studies have investigated the genetic relationships of such a large number of accessions from so many countries. Our study confirmed previous results obtained in a study by the INRAE germplasm collection analyzed with the RosBREED cherry 6K SNP array v1 [15], such as the tight clustering of the Italian accessions associated with ‘Bigarreau Camus de Vénasque’ from Southeast France and ‘Cerna’ from Romania, or the close relationship of two interesting subgroups of landraces maintained in France: the first one consisting of ‘Jerusalem’, ‘Bigarreau Coeur de Pigeon tardif,’ ‘Xapata’, ‘Bigarreau Noir d’Ecully’, ‘Bruelles’, and ‘Blancale Précoce’ and the second of ‘Cristobalina’, ‘Saint Georges’, and ‘Durette’. Our work agreed too with the high proximity highlighted between ‘Napoleon’, ‘Bigarreau Marmotte’, ‘Bing, ‘Sue’, and ‘Nanyo’. Finally, here the narrow genetic relationships of some ‘bigarreaux’ was confirmed: ‘Bigarreau Moreau’, ‘Glacier’ (an offspring of ‘Bigarreau Hâtif Burlat’), ‘Bigarreau Grand’, and ‘Bigarreau Maria Gaucher’ were found close to the popular ‘Bigarreau Hâtif Burlat’ (often known shortly as ‘Burlat’). As previously reported [15], ‘Bigarreau Coeur’ and ‘Bigarreau Saint Bruno’ were associated in another different cluster. Recently, exploring a large collection of genetic resources from fifteen countries, the proximity of ‘Tĕchlovan’ and ‘Hedelfinger’, ‘Emperor Francis’ and ‘Van’, ‘Kassins Frühe’ and ‘Knauffs Schwarze’, and ‘Pivka and ‘Drogans Gelbe Knorpelkirsche’ was shown [28], and all these results agree with our work. At the contrary, the close relationship reported between ‘Libĕjovická raná’ and ‘Kassins Frühe’, ‘Early Rivers’ and ‘Schöne von Marienhöhe’, ‘Pivovka’ and ‘Drogans Gelbe Knorpelkirsche’, ‘Emperor Francis’ and ‘Napoleon’ [28] was not confirmed here.

### 3.4. Implications for Breeding Programs

Our findings demonstrated that a group of sweet cherry landraces were more diverse than a similar-sized group of early selections and modern cultivars. The bottleneck observed in modern cultivars has been well documented for the North American breeding programs [32]. Although the level of co-ancestry and inbreeding has not been recorded in such a precise way for other breeding programs, most of them have used a limited set of key parental genitors, as described in [30]. While this situation is common to numerous agricultural crops and fruit trees, it is particularly challenging to exploit genetic diversity in sweet cherry. Indeed, flowering in sweet cherry is highly dependent on external factors, such as temperature, wind, or humidity (see [33] for a review on sweet cherry flowering biology), which often reduces the number of temporally compatible parents in the field. Another important challenge is the fact that commercially deleterious alleles, such as the ones associated with small fruit [34], appear to be dominant over those associated with large fruit. Hence, landraces, and potentially even more so wild materials, which are characterized by small fruit size, will require multiple backcross cycles to create hybrids with fruit size of commercial value. Despite these hurdles, numerous breeding programs seek to include new alleles conferring higher levels of tolerance/resistance to biotic or abiotic factors, especially within the context of global climate change.

An interesting finding of this study was that landraces, early selections, and modern cultivars co-occurred in most of the main groups determined by STRUCTURE except for K4, which was composed mainly of Northeastern European modern cultivars. Despite a lower genetic diversity within the pool of early selections and modern cultivars, the genetic distances between accessions belonging to these groups appeared to be relatively important. It is highly likely that by carefully selecting popular modern cultivars, including key founders (such as ‘Van’, ‘Bing’, ‘Napoleon’, ‘Emperor Francis’, and ‘Regina’), breeders could maximize genetic diversity in order to avoid the negative effects of inbreeding. However, it is also recommended to incorporate landraces with particularly interesting agronomic traits (e.g., ‘Cristobalina’, characterized by self-fertility and low chilling requirements) and/or with a particularly deviating genetic composition (e.g., Italian landrace ‘Malizia’).

## 4. Materials and Methods

### 4.1. Plant Material

Leaf samples from 324 cherry accessions were obtained from a total of 19 countries, including partners in the EUCherry project and collaborators in the COST Cherry network (https://www.bordeaux.inra.fr/cherry/ accessed on 9 September 2021). Accessions were chosen to meet the following criteria: (i) they should, as far as possible, represent original landraces from the country of provenance; (ii) they should exhibit at least one trait of potential interest to sweet cherry breeders, and (iii) they should ideally be accompanied by characterization data. Some popular old cultivars (resulting from early selection in previous centuries), such as ‘Bigarreau Hâtif Burlat’, ‘Hedelfinger’, and ‘Napoleon’, were included as were some modern cultivars released in breeding programs, such as ‘Bing’, ‘Leningraskaja Chornaya’, ‘Sue’, ‘Valerij Tchkalov’, and ‘Van’. Accessions were noted to classify as either: landraces, early selections, or modern cultivars, according to Campoy et al. [15] (Table S9). Country of origin was either provided by the collection curators, by consulting publications and historical records. If the country of origin could not be determined with certainty, the accession was attributed to the country in which it was maintained. Accession names were corrected for a small number of samples in line with the inferred and corrected profile names identified in Ordidge et al. [9].

### 4.2. DNA Extraction and SSR Genotyping

Genomic DNA extraction was performed with the semi-automated workstation Genesis RMP150 (Tecan Männedorf, CHE) following a slightly modified protocol [35]. Eighteen Single Sequence Repeats (SSRs) loci were chosen based on reported and observed strength of amplification, ease of scoring in a preliminary study, reported heterozygosity, number of alleles, and position on the *Prunus* reference map for optimization in multiplexes. These included 11 loci from the ECPGR recommended genotyping set [36], 4 loci used by other groups for cherry fingerprinting [36,37], 2 loci flanking a QTL region involved in fruit weight determinism [38], and 1 genetically linked to a flesh color major gene [39]. Even though it has been shown that these last three SSRs are associated with genes or QTLs related to important agronomic traits, we hypothesized that the phenotypes corresponding to the different alleles in these loci have the same fitness. Furthermore, the analysis was carried out on samples from germplasm collections and not from natural populations. For multiplexing, the 18 loci were divided into groups based on their expected allele size range. Fluorescent dyes (FAM, HEX, NED, and PET; Life Technologies, ThermoFisher, Foster City, CA, USA) were assigned to each locus in order to distinguish loci within multiplexes (Table S10). The PCR reactions were performed using the Type-it^TM^ Microsatellite PCR kit (Qiagen, Hilden, Germany). Finally, only 14 SSRs were kept for the study; they were distributed across the eight cherry linkage groups and were not closely linked (Table S11, [40,41,42,43]). The SSR analysis was carried out in an ABI 3130 Genetic Analyzer (Applied Biosystems, ThermoFisher, Foster City, CA, USA), using ABI GeneScan and Genotyper software for allele sizing and scoring. In each plate, 3 accessions (‘Noble’, ‘Napoleon’, and ‘F12/1’ (maintained at East Malling, UK), belonging to the ECPGR set of approved references), were included in order to harmonize the scoring.

### 4.3. Data Analysis

Two approaches were followed to identify accessions that shared the same multilocus genotype: visual checking and comparison of profiles of the accessions and the ‘Multilocus Matches’ function available in GenAlex 6.503 software [44]. Following identification, only one representative of each group was retained for the subsequent analyses. In addition, following a similar approach to the European Malus community [31,45,46] and in line with the matching groups identified by Ordidge et al. [9], we propose the allocation of a CHerry UNiQue (CHUNQ) code to the genotypes identified in our study. For genotypes previously identified within matching groups through data alignment [9], CHUNQ numbering is based on the numbers previously allocated to matching groups (including a small number of groups specific to the UK samples that were not previously published). In order to avoid the re-use of allocated numbers, the remaining CHUNQ codes we propose would run sequentially from CHUNQ 109 onwards. We expect that further work will allow a more complete allocation of complementary codes across a wider pool of germplasm in the future.

Parameters of genetic diversity, including the average number of observed alleles, polymorphism information content (PIC), observed heterozygosity (H_o_), expected heterozygosity (H_e_), and inbreeding coefficient (Fis, a measure of heterozygote deviation from the Hardy–Weinberg equilibrium) were estimated using Cervus 3.0 software [47]. The first round of analyses was carried out on the entire set of unique accessions. A second round of analyses was then carried out on accessions classified as (1) landraces and (2) early selections + modern cultivars. Alleles were considered rare when they occurred in less than 1% of the accessions, while those present only in a single accession were denoted as private alleles.

Population structure was investigated with two different approaches. First, a Principal Coordinate Analysis (PCoA) was performed using DARwin 6.0.010 software (Dissimilarity Analysis and Representation for Windows) [48]. Second, a Bayesian model-based cluster procedure was carried out with STRUCTURE 2.3.4 [49]. An admixture model with unlinked loci and uncorrelated allele frequencies was used, with a burn-in period of 100,000 steps and 100,000 MCMC (Monte Carlo Markov Chain) iterations, for K populations ranging from 1 to 19. Ten runs for each K value were performed, and the most likely number of clusters was determined with the plateau criterion [49] and the ΔK method [50]. For a given K, we used the run that had the highest likelihood estimate to assign cluster proportions to individuals. The accessions were assigned to a cluster when 80% or more (coefficient of membership >0.8) of their inferred genome belonged to the cluster, while accessions with lower values were considered admixed. However, in the second set of analyses, these admixed accessions were assigned to groups if their membership values were >0.55, in order to have a global picture of the genetic composition of each national collection. In these analyses, only accessions known as ‘originated in the country concerned’ were considered, whereas cultivars from foreign countries were discarded. In addition, taking advantage of options available in STRUCTURE software, the allele frequency divergence among clusters (net nucleotide distance) and the average distances (expected heterozygosity) between individuals in the same cluster were calculated. Finally, to assess the genetic relationships among the accessions of the European collection, a dissimilarity matrix with a simple-matching index was calculated with 10,000 bootstraps and transformed into Euclidean distances. The Un-Weighted Neighbor-Joining (N-J) method was then applied to the Euclidean distances to build a tree with all genotypes, using DARwin 6.0.010 software [51].

## 5. Conclusions

To our knowledge, the present study, although preliminary, is the largest genetic diversity and structure analysis of combined sweet cherry germplasm from across Europe. Among the samples selected from collections, some new supposed redundancies were identified, and others were confirmed. However, mislabeling and synonymy were not sufficient to explain redundancies concerning some accessions which differ in the country (and sometimes continent) of origin, status, and period of release. A large genetic diversity was found in our collection, in particular within the landrace pool, justifying the efforts made over decades for their conservation. The availability of new sources of diversity should help to address the new challenges that producers face, such as the changing climate and the need to develop more sustainable production systems. However, we would suggest that the picture we present can be further improved with more exhaustive sampling in some of the countries studied and in some of the countries not included, particularly those important in the evolutionary history of the species, including the supposed area of origin of sweet cherry, within the Caucasus region.

## Figures and Tables

**Table 1:** Genetic diversity estimations by 14 loci.

SSR Locus	Na	Allele Range	Rare Alleles	PIC	H_o_	H_e_	Fis	HW
BPPCT034	15	214–258	6 ^# & ##^	0.786	0.95	0.81	−0.17	***
BPPCT037	10	127–170	2 ^#^	0.799	0.85	0.82	−0.04	NS
CPPCT006	12	173–203	4 ^#^	0.750	0.78	0.78	0	NS
CPPCT022	9	245–259	2 ^# & ##^	0.626	0.64	0.68	0.06	NS
CPSCT038	6	184–203	1 ^#^	0.570	0.63	0.63	0	NS
EMPa002	5	105–131	3 ^##^	0.363	0.55	0.46	−0.2	NS
EMPa004	11	160–212	6 ^# & ##^	0.678	0.73	0.73	0	NS
EMPa017	9	229–246	5 ^# & ##^	0.358	0.35	0.38	0.08	NS
EMPa018	14	82–119	8 ^# & ##^	0.640	0.61	0.68	0.1	NS
EMPaS02	8	134–152	1	0.775	0.77	0.8	0.04	NS
EMPaS06	12	203–229	4 ^#^	0.838	0.85	0.85	0	NS
EMPaS12	10	112–155	5 ^# & ##^	0.733	0.79	0.77	−0.03	NS
EMPaS14	7	168–213	3 ^# & ##^	0.558	0.65	0.63	−0.03	NS
PAV-Rf-SSR	9	343–363	3 ^#^	0.737	0.78	0.77	−0.01	NS
Total	137							
Mean	9.8			0.658	0.71	0.70	−0.01	

Na: number of alleles per locus; Private alleles: ^#^ occurring only in one accession; ^##^ occurring only in one collection PIC: polymorphism information content, Ho: observed heterozygosity, He: expected heterozygosity, Fis: inbreeding coefficient, HW: significance of deviation from Hardy–Weinberg equilibrium: NS = not significant, ***: significant at the 0.1% level.

**Table 2:** Genetic diversity estimations in ‘Landraces’ and ‘Early Selections and Modern Breeding Cultivars’.

Collection	N	Na	Rare Alleles	Private Alleles	PIC	H_o_	H_e_	Fis
Landraces	111	9.1	65	16	0.661	0.70	0.70	0.01
Early Selections and Modern Breeding Cultivars	104	7.3	29	8	0.639	0.72	0.69	−0.02

N = number of accessions; Na: number of alleles per locus; PIC: polymorphism information content, H_o_: observed heterozygosity, H_e_: expected heterozygosity, Fis: inbreeding coefficient.

**Table 3:** Allele frequency divergence among clusters (net nucleotide distance).

Cluster	K1	K2	K3
K1	-	-	-
K2	0.1004	-	-
K3	0.1835	0.1178	-
K4	0.1303	0.0996	0.1920

## Data Availability

The SSR genotyping data are provided as supplemental information (see Table S12 in the Supplementary Materials section) in this manuscript.

## Supplementary Materials

The following are available online at https://www.mdpi.com/article/10.3390/plants10101983/s1. Figure S1: Distribution of type of accessions (unique or duplicate genotype) according to the country maintaining the collection, Figure S2: Distribution of private alleles/accessions/collection, Figure S3. Graphical method (as in [48]) allowing the detection of the number of groups K using (a) the rate of change in the likelihood distribution (Mean log-likelihood values), and (b) the ∆K, Figure S4: Genetic composition of the national collections according to the K = 4 populations inferred by STRUCTURE, Table S1: Groups of accessions with identical genotype, Table S2: List of the 220 accessions finally studied including their name, their code, the country which maintained the accession, the country of origin when known, the status of the accession (level of breeding), and the pedigree when known, Table S3: List of accessions showing rare and private alleles in the European collection, Table S4: Genetic diversity estimations (locus per locus) in the landraces and early selection-modern breeding sets, Table S5: Table summarizing the results using the method described in [48] (output of Structure Harvester), Table S6: List of the 220 accessions including membership values to clusters inferred with STRUCTURE, Table S7: Average distances (expected heterozygosity) between individuals in the same population (STRUCTURE output), Table S8: Distribution of the accessions in the N-J Tree clusters, Table S9: List of accessions studied (314 accessions), including their name, their code, the country which maintained the accession, the country of origin when known, the status of the accession (level of breeding, according to [16]) and the pedigree when known, Table S10: Composition of initial SSR panel used for genotyping, Table S11: List of SSR finally studied and their characteristics, Table S12: Raw data (314 accessions).
