# Supplementary material for: SSR-Based Analysis of Genetic Diversity and Structure of Sweet Cherry (Prunus avium L.) from 19 Countries in Europe
Source: Plants (Basel). 2021 Sep 23;10(10):1983. doi: 10.3390/plants10101983 (PMC8540667; doi:10.3390/plants10101983)
Supplement: Supplementary file 1 [file plants-10-01983-s001.zip › Cherry genetic diversity_Barreneche et al_Supplementary file Figures S1-S4-revised.pdf]

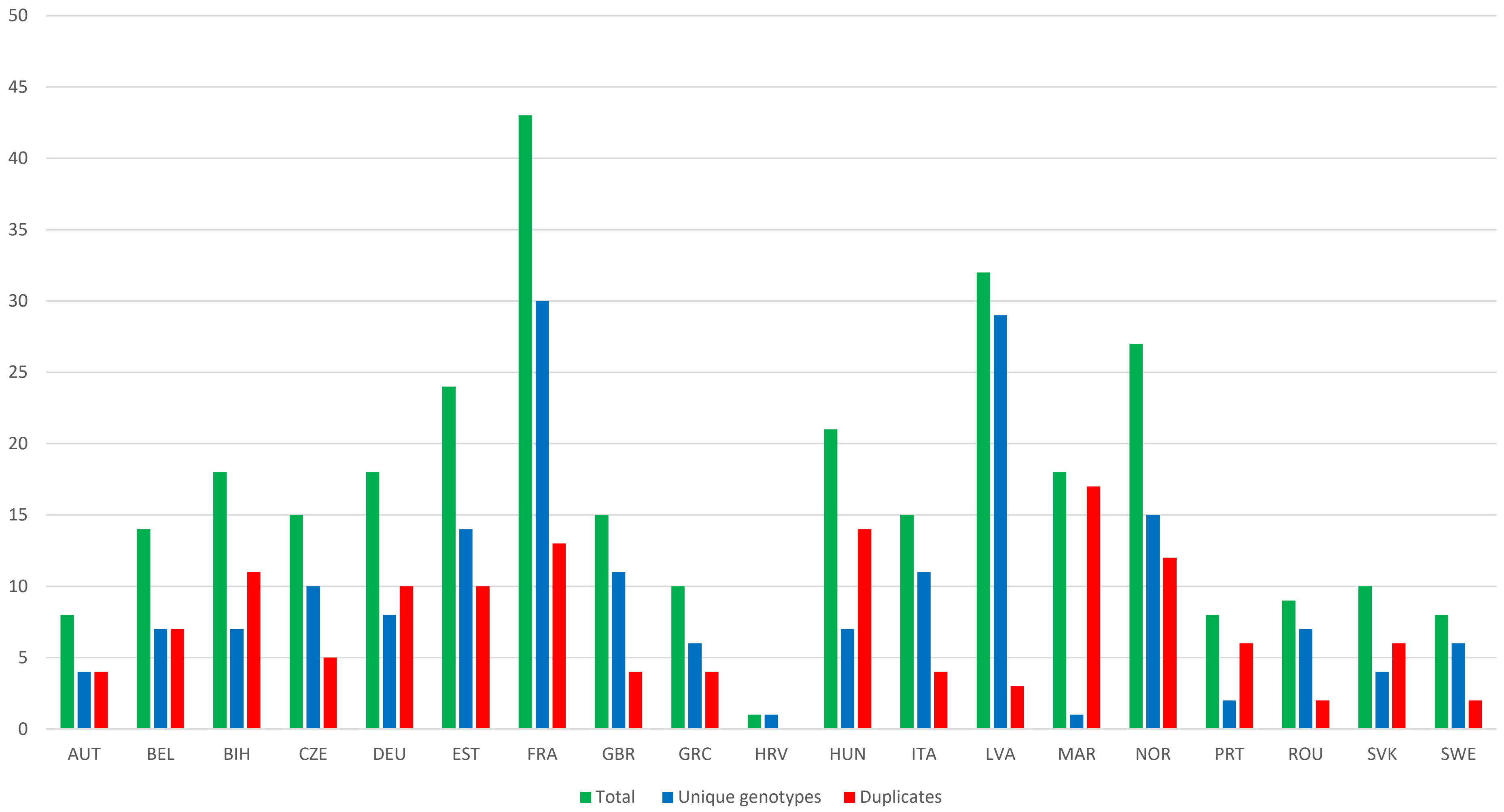

Figure S1: Distribution of type of accessions (unique or duplicate genotype) according to the country maintaining the collection

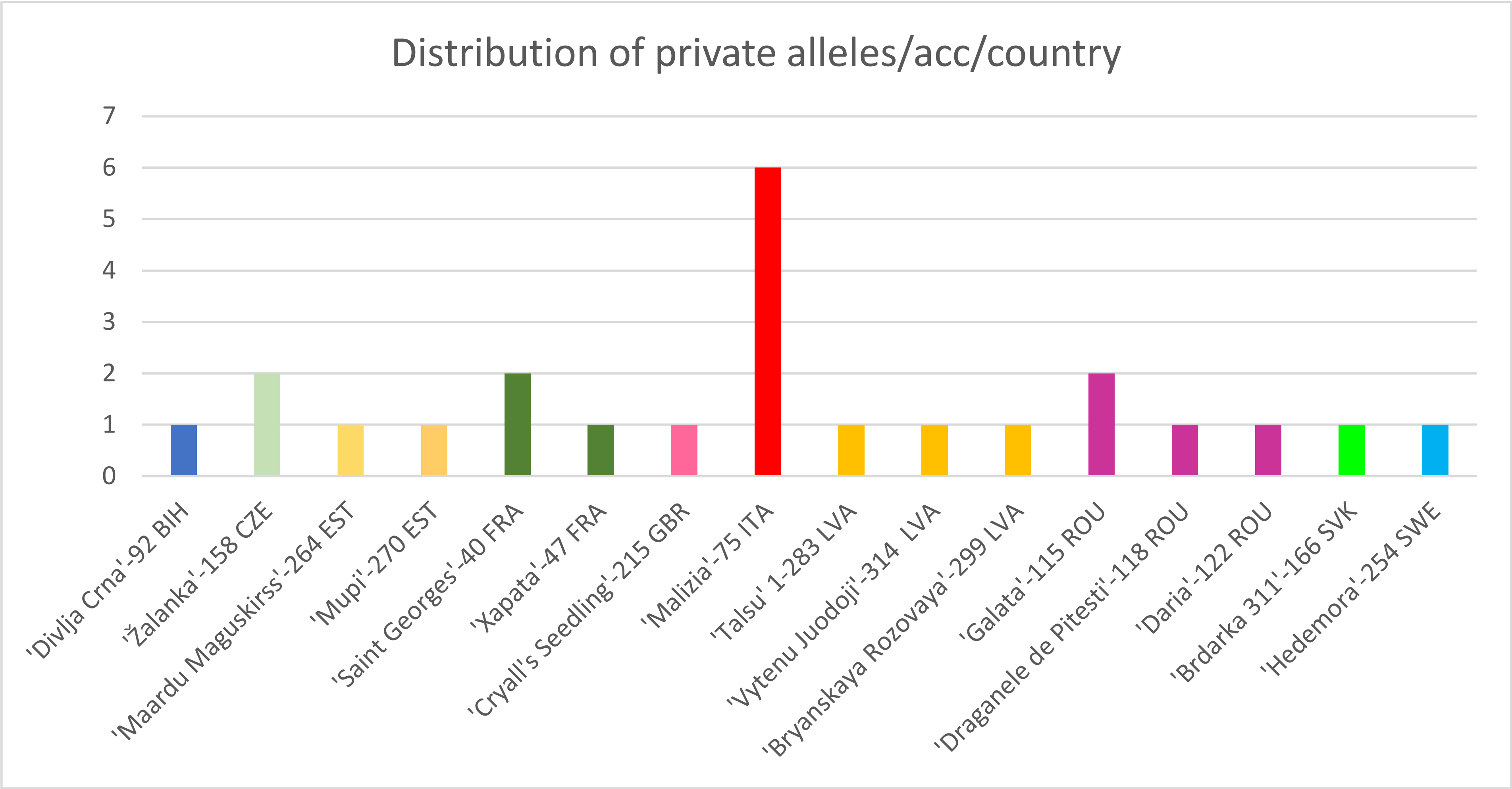

Figure S2: Distribution of private alleles/accessions/collection

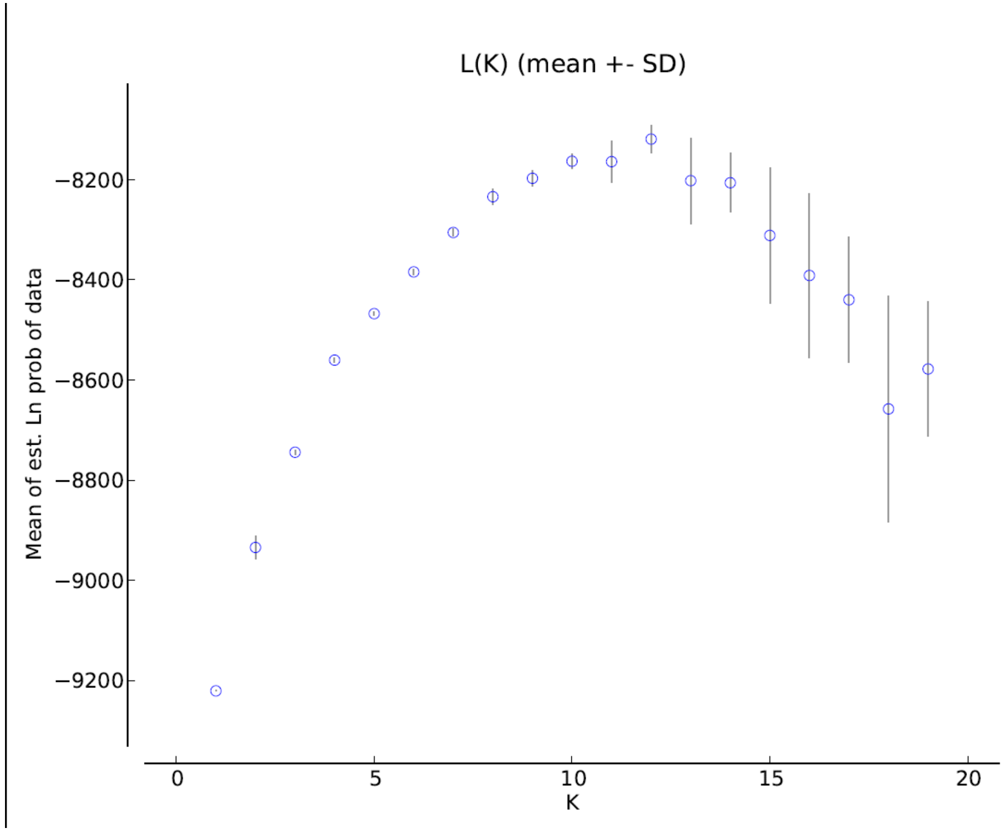

a

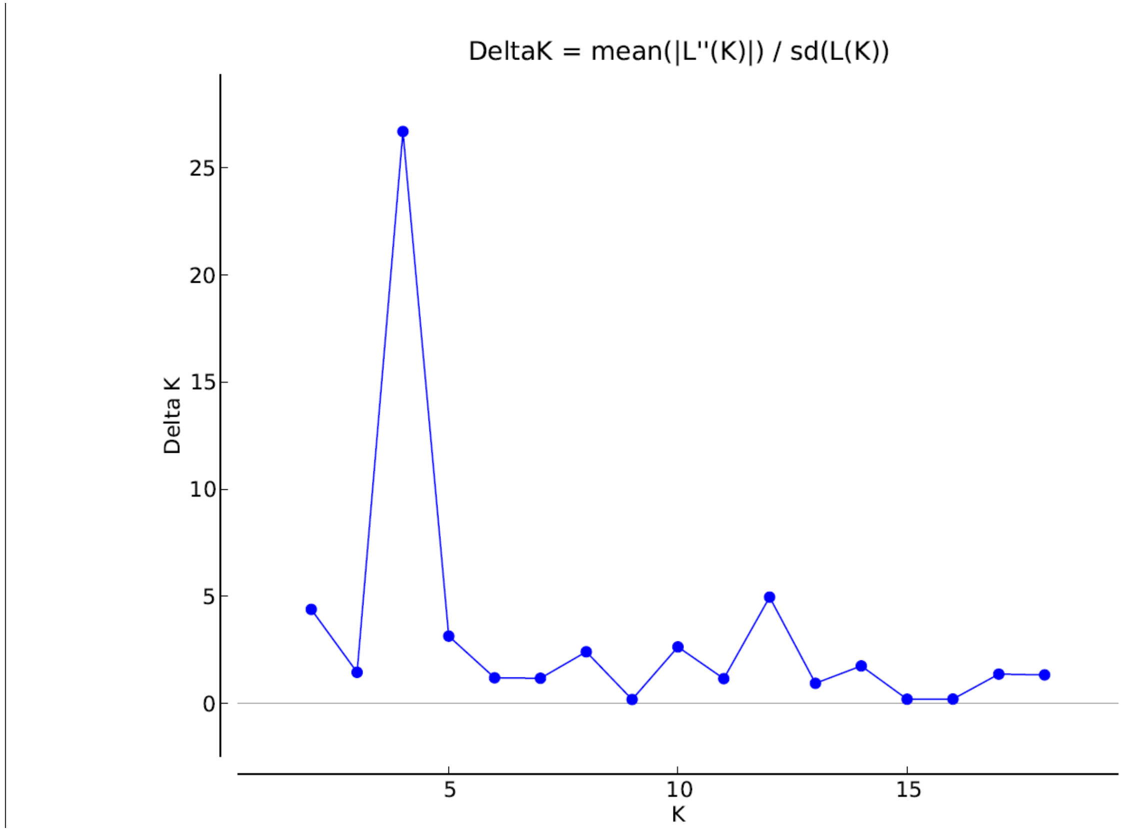

b

Figure S3: Graphical method (as in [48]) allowing the detection of the number of groups K using: (a) the rate of change of the likelihood distribution (Mean log-likelihood values) and (b) the ΔK

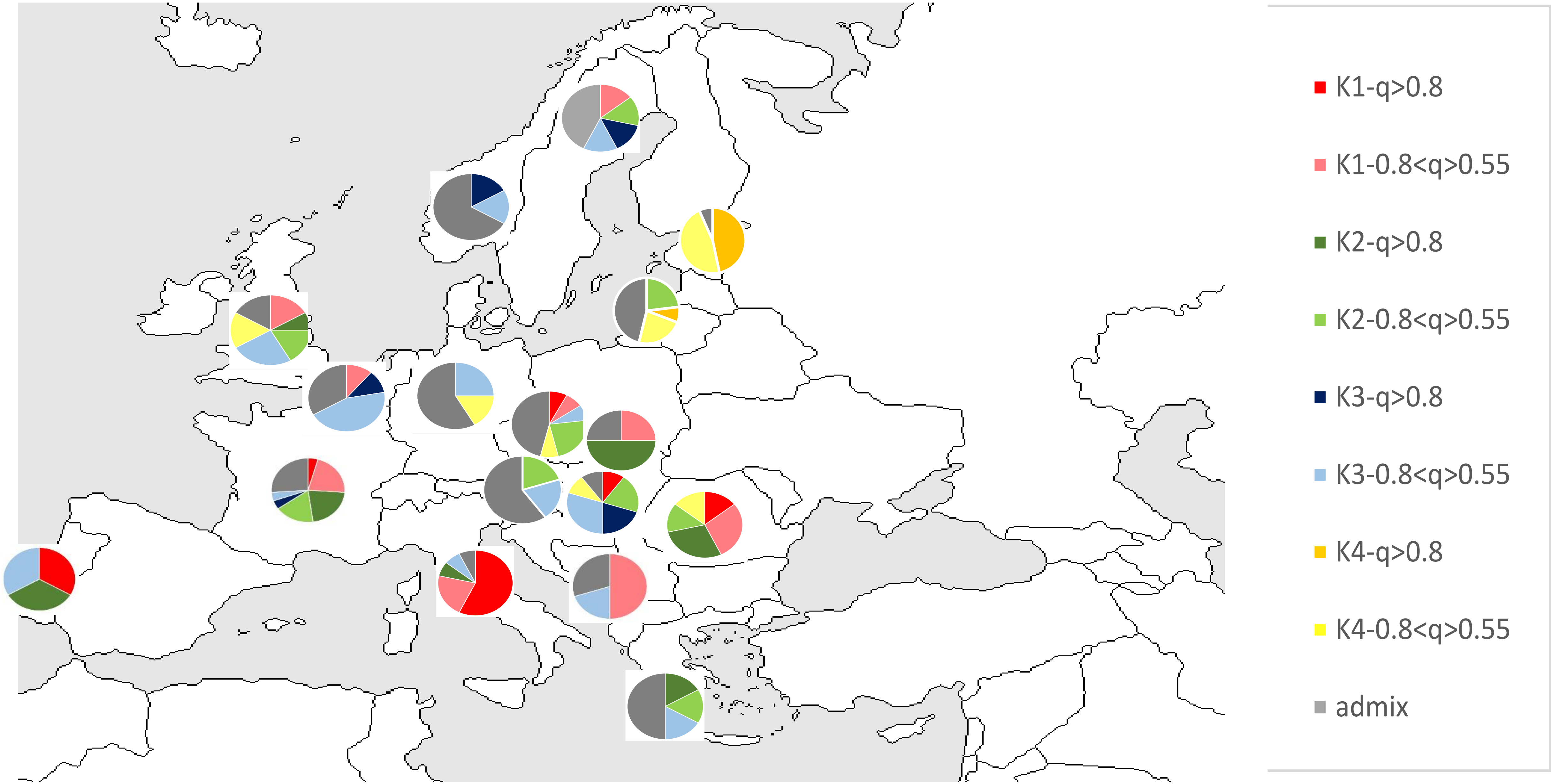

Figure S4: Genetic composition of the national collections according to the K= 4 populations inferred by STRUCTURE.
